# An Integrated TDR Waveguide and Data Interpretation Framework for Multi-Phase Detection in Soil–Water Systems

**DOI:** 10.3390/s25154683

**Published:** 2025-07-29

**Authors:** Songcheng Wen, Jingwei Wu, Yuan Guo

**Affiliations:** State Key Laboratory for Tunnel Engineering, School of Civil Engineering, Sun Yat-Sen University, Guangzhou 510275, China; wensch@mail2.sysu.edu.cn (S.W.); wujw225@mail2.sysu.edu.cn (J.W.)

**Keywords:** time domain reflectometry, suspended sediment concentration, water level, riverbed scouring

## Abstract

Time domain reflectometry (TDR) has been validated for monitoring water level evolution and riverbed scouring in the laboratory. Previous studies have also validated the feasibility of field-based single hydrological parameter monitoring using TDR. However, the current research focuses on developing separated TDR sensing systems, and integrated measurements of multiple hydrological parameters from a single reflected waveform have not been reported. This study presents an improved helical probe sensor specifically designed for implementation in geologically hard soils, together with an improved data interpreting methodology to simultaneously determine water surface level, bed elevation, and suspended sediment concentration from a single reflection signal. Experimental comparisons were conducted in the laboratory to evaluate the measuring performance between the traditional dual-needle probe and the novel spiral probe under the same scouring conditions. The experiments confirmed the reliability and superior performance of spiral probe in accurately capturing multiple hydrological parameters. The measurement errors for the spiral probe across multiple hydrological parameters were all within ±10%, and the accuracy further improved with increased probe embedding depth in the sand medium. Across all tested parameters, the spiral probe showed enhanced measurement precision with a particularly significant improvement in suspended sediment concentration detection.

## 1. Introduction

Modern near-water infrastructure is encountering increasing challenges under extreme climate events, such as flooding and storm surge with rapidly rising water level accompanied by intensified hydrodynamic forces [1,2]. The fluctuation in water level combined with different hydrodynamic factors enhance shoreline erosion processes and sediment transport in water bodies [3], which undermine the stability of hydraulic facilities and potentially lead to partial collapses or structural failures [4,5,6]. Monitoring of these hydrological factors is essential for the life-span maintenance of sustainable infrastructure [7,8].

Water level, sediment concentration, and riverbed scour are three major components in assessing hydrodynamic forces on structures. It is generally reported the fluctuating water level causes varying flow velocity profiles and is often accompanied by changing erosion process for the riverbed morphology [9,10]. The associated local scour of foundations is regarded as one of the main causes for facility failures [11]. Furthermore, sediment transport is another critical aspect determining the riverbed deposition and erosion process, in which the concentration of suspended sediments has been utilized as a major indicator [12,13]. Particularly during extreme climate events, the rapid increase in hydrodynamic forces leads to much higher sediment concentrations and riverbed erosion activities [14,15,16].

The current monitoring devices are easily affected by harsh environments in terms of their applicability and detection accuracy and are installed separately with a high maintenance cost. TDR only requires the deployment of a metal waveguide probe at the target location, while other electronic components can be placed in a secure area. The metal probe is cost-effective and highly resistant to damage [17]. Water level monitoring during extreme weather events provides key information for assessing the impact and risk level of hydrological events [18]. Various instruments can be used for water level and riverbed elevation measurements, including sonar and radar. However, these sensors usually need to be integrated as a whole system, and the sonar detection requires an optimal sediment concentration range. The sediments can be classified into suspended load and bed load based on their movements [19], and the suspended load, consisting of fine particles, contributes to a significant proportion of sediment transport [20]. Real-time monitoring of suspended sediment concentration is usually achieved through fluid turbidity measurement [21]. However, the proper regression from turbidity to suspended sediment concentration depends on the physical characteristics of particles. Other acoustic and optical instruments also have limitations such as being unable to adapt to a wide detection range or the sensors being prone to damage.

Over the past decades, TDR-based instruments have been developed and implemented in various engineering fields, including extensive applications in hydraulic engineering domains [22,23]. Typical implementations include accurate monitoring of soil moisture profile, real-time water table fluctuation, and in situ suspended sediment concentration characterization [24,25,26]. To increase the measurement resolution, researchers have developed optimized waveguide configuration and advanced analytical frameworks for TDR probe and data interpretation [22,27]. Yu and Yu [28] developed an innovative TDR system specifically tailored for bridge scour depth monitoring and demonstrated sub-centimeter-level accuracy. Furthermore, the dielectric permittivity of electrolytes is significantly influenced by the suspended particles, and there exists a correlation with suspended sediment concentration (SSC) [29,30]. Miyata et al. [24] engineered a multi-height TDR sensing system incorporating adaptive waveguide profiling, thereby validating the technical feasibility of TDR-based SSC measurements with a high sediment concentration. Tidwell and Brainard [31] employed TDR for continuous monitoring and assessment of dynamic river stage fluctuation and bedform morphology. These TDR-based studies focus on measurements of single hydrological parameters without considering the integration of multi-phase detection techniques.

This paper presents our experimental work on the development of integrated TDR probes with an improved data interpreting methodology. Our laboratory results show that the system is able to simultaneously determine water level, bed elevation, and SSC from a single reflection signal, which greatly reduces the field installation and maintenance costs. We also compared the performance of two types of sensing probes, i.e., linear and spiral, in multi-parameter measurements under different conditions. The proposed analytical methods and corrections improve the monitoring accuracy for optimized multi-parameter characterization.

## 2. TDR Principle and Data Interpretation Methodology

The basic principles of TDR system will be discussed in detail in this section. The data interpretation methodology for detecting different interfaces and apparent lengths along with SSC are provided as well.

### 2.1. Basic Principle of TDR Systems

TDR systems measure the electromagnetic waves propagating along cables or metallic sensing waveguides and have been used in the communication industry for cable discontinuity testing [32]. When electromagnetic waves propagate along a transmission line and encounter impedance discontinuities, which represent energy imbalances in the field, signal reflections occur [33,34]. The system consists of three main parts, i.e., the pulse generator, the wave signal sampler, and the transmission line. A pulse generator launches electromagnetic waves into the transmission line system, while a signal sampler records the reflected signals [35,36]. By analyzing the propagation time and characteristics of the reflected waveform, variations in the spatial distribution of materials can be determined.

TDR relies on the determination of propagation velocity of electromagnetic waves along parallel metallic probes embedded in the medium of interest. The fundamental physical property affecting the pulse transit time is the dielectric property of the medium along with the propagation velocity. The travel time of the electromagnetic wave depends on the dielectric constant *K_a_* of the material around the probe, as shown in Equation (1). All dielectric constants mentioned in this paper refer to relative dielectric constants, defined as the ratio of a material’s dielectric constant to that of a vacuum.

(1) $$K_{a}={\left(\frac{c\Delta t}{2L}\right)}^{2}={\left(\frac{L_{a}}{\upsilon_{p}L}\right)}^{2}$$

where Δ*t* is the two-way travel time of the electromagnetic wave propagating in the transmission line; *c* is the velocity of the electromagnetic wave, which is equal to light speed in a vacuum (2.99 × 10^8^ m/s); *L_a_* is the apparent length of the probe; *L* is the actual probe length; *ν_p_* is the propagation velocity coefficient of the transmission line.

The reflection coefficient *ρ* is usually used to quantify signal amplitude, which is defined as the ratio of the reflected signal amplitude to the incoming signal amplitude, see Equation (2). When an immersed transmission line is connected to a TDR system with an output impedance *Z*_0_, the mismatched impedance of *Z*_0_ versus *Z_L_* causes part of the input signal to be reflected to the TDR signal sampler, while the remainder of the signal is transmitted into the sample.

(2) $$\rho =\frac{V_{re}}{V_{in}}=\frac{Z_{L}-Z_{0}}{Z_{L}+Z_{0}}$$

where *V_re_* is the reflected voltage amplitude; *V_in_* is the incident voltage amplitude; Z*_L_* is the output impedance of the TDR cable tester; *Z*_0_ is the uniform characteristic impedance.

Experimental results demonstrated that for the designed 50 Ω matched coaxial probes filled with the sample under testing, the dielectric change at the air–dielectric material interface can be related to the reflection coefficient through the following equation [37].

(3) $$K_{a}={\left(\frac{1-\rho }{1+\rho }\right)}^{2}$$

### 2.2. Data Interpretation for Interface Detection

Three phases exist in a typical fluvial soil–water system, including saturated bed materials, water with suspended sediment, and free air. A sediment suspension is mainly composed of water and solid particles. The apparent dielectric constant of soil particles *K_s_* is temperature-insensitive with a narrow range from 3 to 9, depending on their mineral composition [38]. On the contrary, the dielectric constant of water *K_w_* is around 80 and temperature-dependent, as indicated in empirical Equation (4) [39]. As a result, the temperature is a critical parameter for calculating the water dielectric constant. In the experiments, water temperature was obtained using temperature sensors installed near the water surface, with a measurement accuracy of ±0.2 °C. The dielectric constant of air is around 1. Differences in dielectric constant among different phases provide opportunities to detect interfaces among the system with a single reflective signal.

(4) $$K_{w}\left(T\right)=78.54\left[1-4.58\times 10^{-3}\left(T-25\right)+1.19\times 10^{-5}{\left(T-25\right)}^{2}-2.8\times 10^{-8}{\left(T-25\right)}^{3}\right]$$

where *T* is the measured temperature in degrees Celsius.

Yu and Yu [28] developed an algorithm to determine scour depth from TDR signals, which is based on the application of a volumetric mixing model. This study extended the dielectric mixing model to a layered system consisting of a water layer and sediment layer. Comparing to traditional direct measurements of discontinuous layers, this approach would be preferable because of the much lower propagation velocity of the TDR pulse in water than in air (about one over nine). In addition, the direct measurement of water depth requires a preset propagation velocity for sediment suspensions, which is not feasible compared to pure water in the literature.

**Figure 1:**
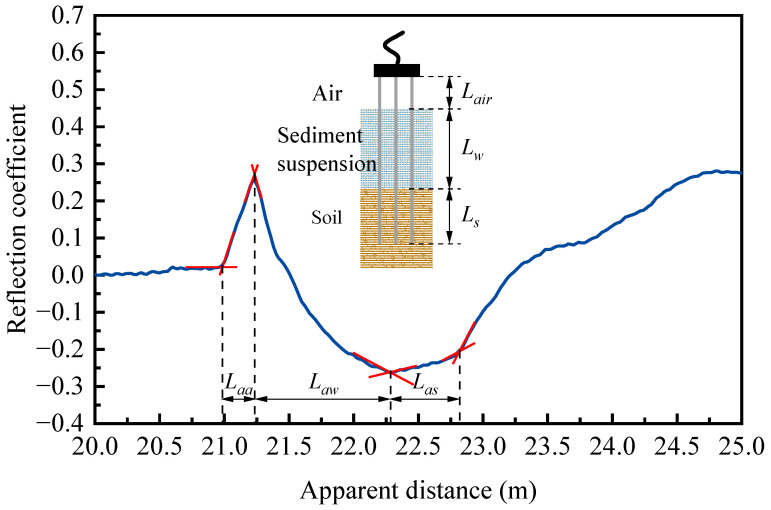
Schematic diagram of division of waveform reflected by different media.

For a TDR probe embedded vertically along the riverbed profile, the measured total travel time of the signal pulse is a summation of the travel times in different phases, including that of the sediment suspension layer and the saturated soil layer, as is shown in Figure 1. The overall reflected waveform is regarded as the superposition of the contributions from the water and saturated sand components. By quantifying the influence of each component on the reflected waveform, the respective thicknesses of the water and sand layers can be determined using Equation (5) [28].

(5) $$\frac{\sqrt{K_{a,m}}}{\sqrt{K_{a,w}}}=\frac{L_{ws}-L_{s}}{L_{ws}}\left(\frac{\sqrt{K_{a,s}}}{\sqrt{K_{a,w}}}-1\right)+1$$

where *K_a_*_,*w*_ is the dielectric constant of the sediment suspension; *K_a_*_,*s*_ is the dielectric constant of the saturated soils; *K_a_*_,*m*_ is the measured bulk dielectric constant of the system consisting of the sediment suspension layer and saturated soil layer; *L_s_* and *L_ws_* are the thicknesses of the soil layer and soil with suspension layer, respectively.

During the test, the electromagnetic signal travels through air, sediment suspension, and saturated soils sequentially and reflects at four points (i.e., coaxial cable–probe, air–suspension, and suspension–soil interfaces and the probe end), as is shown in Figure 1. Along with this segmentation, the positions of water level and soil bed can be determined, enabling an integrated measurement of water depth and scouring distance from a single reflected waveform. The distribution lengths of each medium are calculated based on Equation (6).

(6) $$\left\{\begin{array}{c} L_{air}=\frac{L_{aa}}{\sqrt{K_{a,a}}}\\ L_{s}=\frac{L_{as}}{\sqrt{K_{a,s}}}\\ L_{w}=L-L_{air}-L_{s} \end{array}\right.$$

where *L_aa_* represents the apparent length of reflection segment in air; *L_as_* is the apparent length embedded in saturated soils; *L_w_* is the physical length of the probe within the suspension; *K_a,a_* and *K_a,s_* refer to the dielectric constants of air and saturated soil, respectively.

### 2.3. Data Interpretation for SSC Characterization

According to the dielectric mixing theory, the bulk dielectric permittivity of sediment suspension can be expressed as a function of SSC by the volumetric mixing model, as is shown in Equation (7).

(7) $$\sqrt{K_{a}}=\left(1-\theta_{s}\right)\sqrt{K_{w}\left(T\right)}+\theta_{s}\sqrt{K_{ss}}$$

where *K_a_* is the bulk apparent dielectric constant of the sediment suspension; *θ_s_* is the volumetric SSC ranging from 0.0 to 1.0; *K_ss_* is the apparent dielectric constant of the suspended particles.

Once *K_w_*(*T*) and* K_ss_* are given, the volume fraction of sediments *θ_s_* can be determined based on the measured apparent dielectric constant* K_a_* in the sediment suspension in Equation (8) [25].

(8) $$\theta_{s}=\frac{\sqrt{K_{a}}-\sqrt{K_{w}\left(T\right)}}{\sqrt{K_{ss}}-\sqrt{K_{w}\left(T\right)}}$$

Usually, the volume fraction of sediments *θ_s_* would be converted into suspended sediment concentration in g L^−1^, as is shown in Equation (9), which is commonly used in hydraulic engineering.

(9) $$SSC=\frac{\theta_{s}G_{s}}{1-\theta_{s}}\times 10^{3}$$

where *G_s_* is the specific gravity of suspended sediments, which typically ranges from 2.54 to 2.63 for kaolinite particles.

## 3. Experimental System with Probe Fabrication

Following the above methodology, a TDR testing system was set up to analyze the one-stage detection algorithm of water depth, scouring distance, and SSC from a single repulse signal. A spiral-shaped probe was proposed, fabricated, and validated in parallel with conventional dual-rod and three-rod probes.

### 3.1. Experimental Setup and Materials

**Figure 2:**
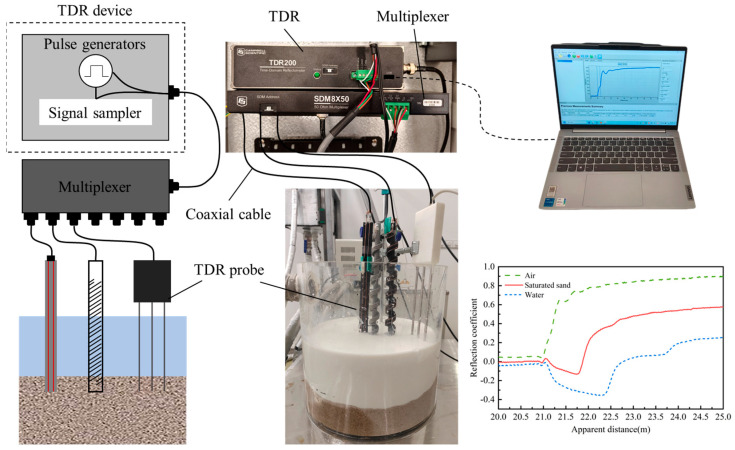
Schematic diagram of main components and connecting sequence of TDR measurement system.

The experimental system consists of a time domain reflectometer and a transmission line with sensing probes. The TDR equipment typically includes a pulse signal generator and an electromagnetic wave signal sampler, while the transmission line system consists of coaxial cables that transmit electromagnetic waves and waveguide probes placed in the medium for testing. A single TDR equipment can be connected to multiple probes simultaneously through a multiplexer, as shown in Figure 2. The TDR device generates an electromagnetic pulse that propagates through the transmission line system, and captures reflection signal data. This reflected signal is related to the material properties and impedance characteristics around the probe. The typical experimental setup is composed of a TDR miniaturized unit (TDR200, Campbell Scientific, Logan, UT, USA), processing control software PC-TDR v3.0, coaxial cables, different types of probes, and a multiplexer (SDM8X50; Campbell Scientific), working like closed-circuit radar and detecting any mismatch along the measuring lines. During the testing, pulse signals are generated from TDR and transmitted into different coaxial cables in sequence to allow multi-probe monitoring within a few seconds. The excited TDR signal is a step electromagnetic impulse, which is characterized by a rise time of 200 ps, corresponding to a frequency bandwidth of around 2.75 GHz.

**Figure 3:**
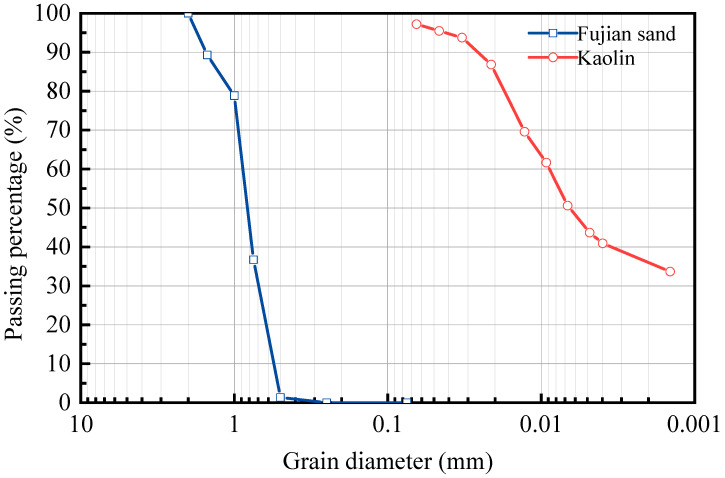
Particle size distribution of Fujian sand and kaolinite clay.

To ensure a precise environmental control, standard Fujian sand was used as the saturated bed material, and SSC was prepared by mixing industrial kaolinite clay with pure water. Particle gradation of sands and clay were firstly analyzed by sieve and hydrometer tests. Figure 3 illustrates the particle size distributions of Fujian sand and kaolinite clay. During the TDR testing, a small electromagnetic stirrer was used to continuously agitate the sediment suspension in order to maintain a uniform suspended state while avoiding sand bed disturbance. Both Fujian sand and kaolinite were assigned a dielectric constant of 5.0 for the following analysis, and the studied range of SSC is 40–120 g/L. During TDR probe deployment, the probe must be embedded across all three media, including air, suspension and sand, to ensure electromagnetic wave signals propagate along the probe simultaneously to detect both water surface and riverbed elevations.

### 3.2. Design of Innovative TDR Probe

The measuring resolution of the reflected waveform is influenced by the rise time of the TDR signal and the length of the waveguide. Since it is difficult to adjust or improve the rise time of the TDR signal due to device limitations, using a longer waveguide can effectively enhance the measuring resolution of the medium. A helical probe structure allows for a longer waveguide to be arranged within the same probe length, thereby significantly improving measuring resolutions [22,24,40]. The conventional helical probes typically adopt a structure where metal wires are wound around the outer side of a cylindrical support rod. The metal wires lie closely along the supporting structure, making them prone to displacement when inserted into the medium, which inevitably undermines probe accuracy, especially in field conditions. On the other hand, for hard and compact soil layers, the large cylindrical structure is rather difficult for installation due to lateral repelling of surrounding soils.

**Figure 4:**
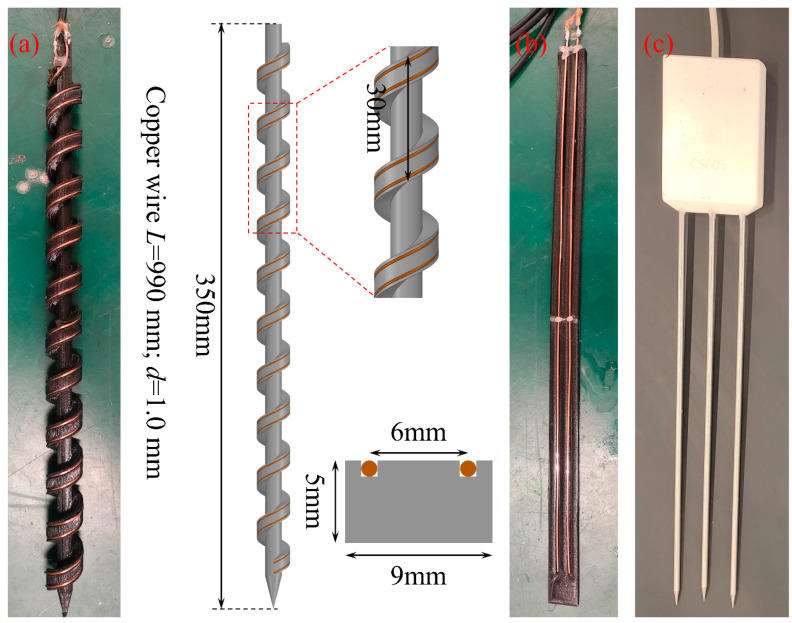
Comparison of three different TDR probes: (**a**) new spiral-shaped TDR probe (in mm); (**b**) lab-made conventional two-rod probe; (**c**) commercial three-rod TDR probe.

In this study, the traditional helical probe with wires laid on the cylindrical surface was improved by changing its supporting configurations. Figure 4 presents the customized TDR probe with comparisons of two conventional dual-rod and three-rod probes. The new helical probe consists of a central supporting rod, helical blades, and two metal wires. These components were incorporated into an integrated spiral auger-shaped structure and fabricated using a 3D printer with polylactic acid (PLA) for subsequent laboratory testing. The two metal wires are embedded in the predefined grooves with 6 mm separating distance on the spiral auger structure. Our design configurations have been shown to be effective in preventing wire displacement during the probe installation and significantly reducing the driving resistance for deployment into hard soil layers.

It should be mentioned that the probe design parameters were determined based on comprehensive considerations of measuring accuracy, ease of installation, and fabrication. Although increasing the space between the helical blades will improve probe’s drivability into hard soils, it reduces measuring accuracy as a result of affecting the total wire length. The distance between two wires was determined based on the electric properties of the testing media and needs to be adjusted for future larger probes used in field conditions. A dual-wire configuration was adopted with two bare copper wires wound in parallel. Each wire is 960 mm long and 1.0 mm in diameter. The diameter of the rod was designed to balance structural strength for installation and the minimization of skin effect for soil disturbance. The spiral probe was compared with two other types of probes, i.e., a conventional dual-rod probe with the same wire configurations and a commercial three-rod probe, as is shown in Figure 4.

### 3.3. Probe Calibration and Validation

The transmission wires of the spiral-shaped probe are embedded within the prefabricated grooves of the helical blades made of PLA material. As the electric properties of PLA are different from those of the testing media, it is necessary to conduct probe calibration to minimize the influence of the spiral support structure on TDR signal response. The dielectric constant measured by the probe is jointly determined by the dielectric properties of the surrounding soil–water medium and the PLA structural material itself. The influence of these two factors on the measured dielectric constant *K_am_* can be expressed by the following equation.

(10) $$\sqrt{K_{am}}=\alpha \sqrt{K_{PLA}}+\left(1-\alpha \right)\sqrt{K_{m}}$$

where *α* is the influential coefficient of the spiral structure on measured dielectric constant; *K_PLA_* is the dielectric constant of the spiral structure material (i.e., polylactic acid); *K_m_* is the dielectric constant of the soil–water medium surrounding the probe.

**Figure 5:**
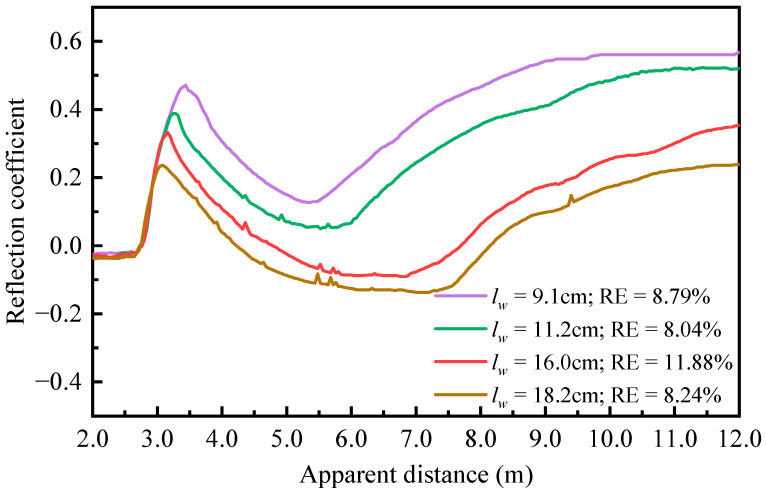
Reflection signals obtained from the spiral-shaped probe at different water levels.

A series of reflection waveform measurements were conducted in deionized water and air to determine the influential coefficient *α*. For a given probe, the value of α will remain constant, and the experimental results determined that the value of α for the customized helical structure falls within the range of 0.257 to 0.244. A final value of 0.251 has been used in the following analyses. The accuracy of the probe measurements was subsequently validated by adjusting the water level. In the validation experiment, the spiral-shaped probe was fixed inside a large acrylic cylinder, as shown in Figure 2. The position of the spiral-shaped probe was maintained constant, while the water depth was adjusted at four different levels. For each case, three parallel measurements were performed, and the water level was inspected visually using a ruler. The measuring performance of the new spiral-shaped probes is shown in Figure 5. To further consider the influence of environmental temperature, the dielectric constant of deionized water has been corrected based on ambient temperature according to Equation (4). The fabricated spiral-shaped probe demonstrates a high monitoring accuracy with simple installation procedures.

## 4. Results and Discussion

We further evaluate the influence of fluid temperature on measuring accuracy based on corrected dielectric constants. The performance of the spiral-shaped probe is subsequently compared with conventional dual-rod and three-rod probes in terms of interface and SSC detections, demonstrating its superior performance.

### 4.1. Temperature Effect on Hydrological Measurements

**Figure 6:**
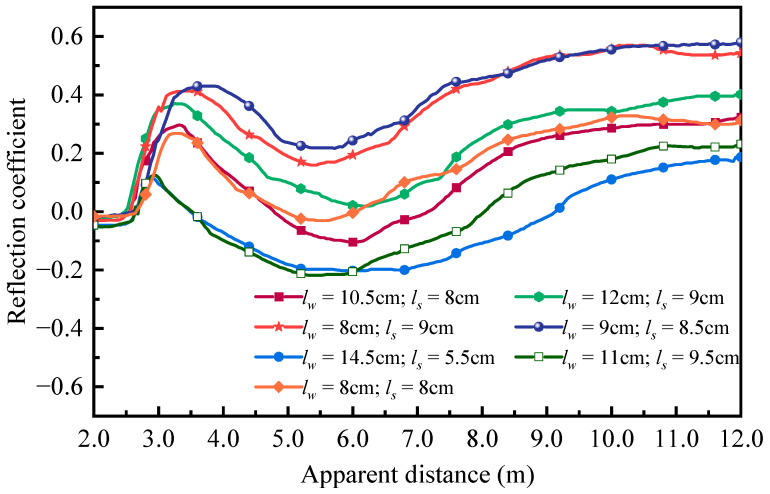
TDR waveforms under different water surface and sand bed elevations using spiral probe at 20 °C.

The TDR system was further evaluated by conducting experiments under varying testing conditions, including changing water depths, bed elevations, and suspended sediment concentrations. The environmental temperature affects the dielectric constants of the soil–water system and, in return, determines the measuring accuracy of the TDR system. To validate the proposed temperature correction algorithm, TDR experiments were conducted on varying temperature conditions using three types of probes. The baseline temperature was set at 20 °C, and the water temperature was gradually increased to 24.1 °C and 35.3 °C through a water bath unit. The sensing probes were connected to the TDR device via the SDM8X50 interface. Figure 6 presents the TDR reflection waveforms recorded by the spiral probe under different water depths *l_w_* and sand bed elevations *l_s_* under 20 °C temperature.

**Figure 7:**
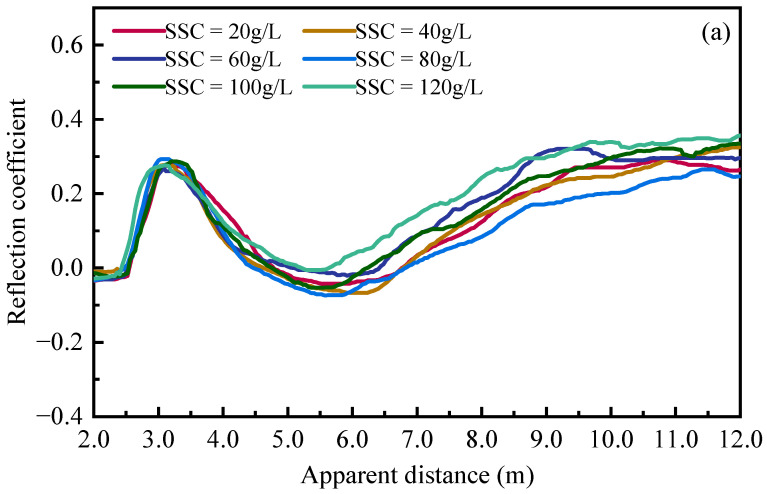

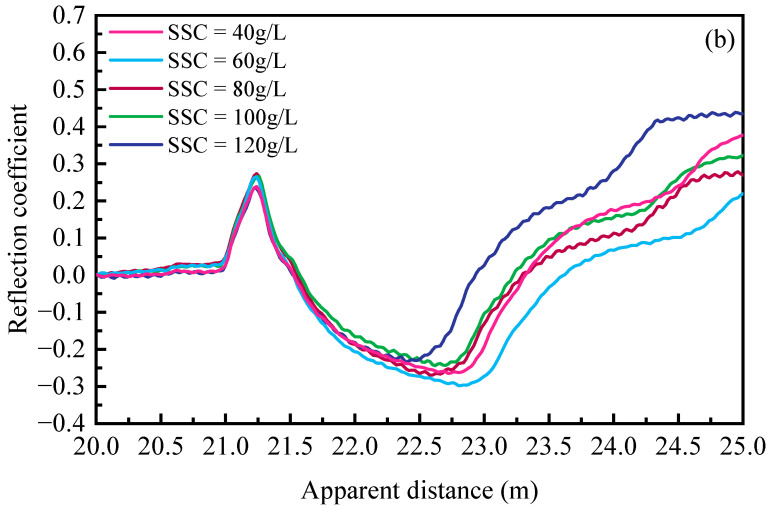
TDR waveforms under different suspended sediment concentrations at 20 °C: (**a**) customized spiral probe; (**b**) commercial three-rod probe.

Figure 7 plots the measured reflected waveforms under different suspended sediment concentrations using the customized spiral probe and commercial three-rod probe at 20 °C. It clearly indicates that, as the water surface and sand bed elevation remain unchanged, the increasing SSC only affects the reflection segment corresponding to the water–air interface. With the increase in SSC in the fluid phase, the dielectric constant of suspensions decreases due to the existence of kaolinite particles, leading to a significant reduction in the apparent length of fluid segment in the reflected signal. Changes in the apparent length could be used to quantify sediment concentrations using the mixture model with a decent accuracy.

**Figure 8:**
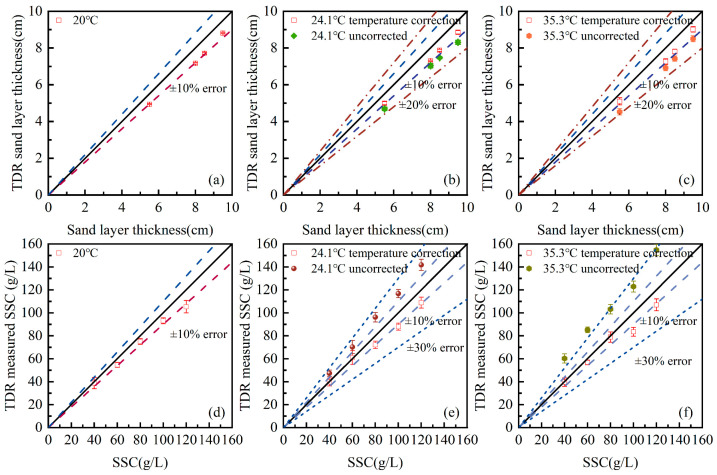
Temperature-related correction for TDR post-analysis on measuring sand bed thickness and suspended sediment concentration: (**a**) sand bed thickness at 20 °C; (**b**) sand bed thickness at 24.1 °C; (**c**) sand bed thickness at 35.3 °C; (**d**) SSC at 20 °C; (**e**) SSC at 24.1 °C; and (**f**) SSC at 35.3 °C.

As discussed in Section 2.2, the dielectric constant of water is highly associated with temperature, which, in return, affect the calculated elevations of water–air and air–sand interfaces. The temperature effect is generally ignored in the previous data interpretation approaches. Here, the changing dielectric constant has been implemented in the proposed algorithm to minimized post-analysis errors related to the environmental temperature. Figure 8 compares the final results of temperature-related corrected and uncorrected approaches under 20 °C, 24.1 °C, and 35.3 °C. The measuring parameters include sand bed depth and SSC.

The uncorrected approach uses the dielectric constant of deionized water at 20 °C in the post-analysis, and as a result, no correction is needed for the 20 °C cases. For testing cases under a higher environmental temperature, the dielectric constant of water decreases based on Equation (4), while the dielectric constant of sands maintains relatively stable. A decrease in dielectric constant leads to a larger layer thickness in the temperature-corrected approach, as is shown in Figure 8b,c. The uncorrected approach predicts sand layer thickness at errors between 10% to 15%, while the corrected values all lie in 10% at 24.1 °C and 35.3 °C. For the SSC measurement, the uncorrected approach assigns higher values to the dielectric constant for the soil–water system under high temperatures, which leads to overestimations of suspended sediment concentration, as is shown in Figure 8e,f. The temperature-corrected approach is able to predict good values, especially for lower SSC conditions (≤80 g/L), and most of the errors lie in 8%.

### 4.2. Detections on Water and Sand Bed Elevation

**Figure 9:**
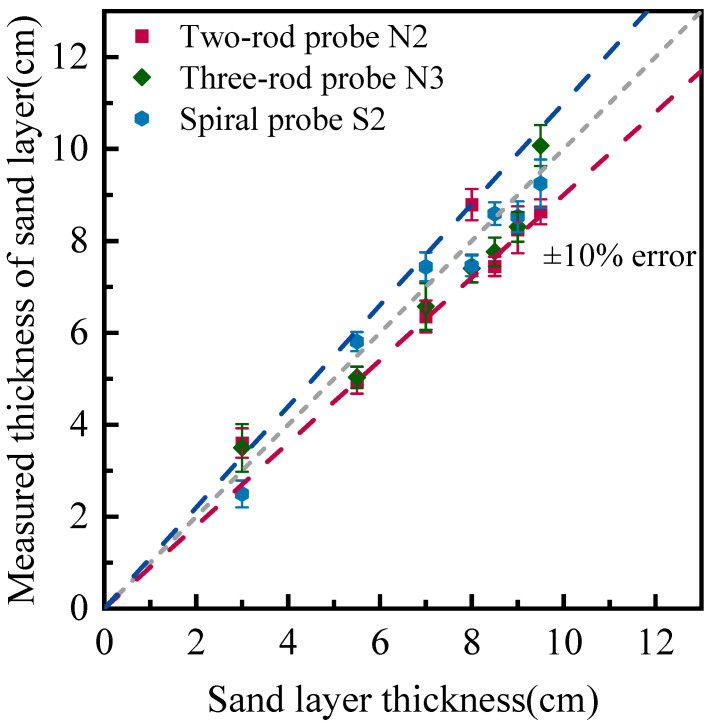
Measurement of sand bed elevation using different types of probes.

The water depth and sand bed elevation can be determined by analyzing reflective signals at the air–suspension and suspension–sand interfaces caused by the discontinuity of the dielectric constant of materials. The accurate measurement of water and sand bed surfaces relies on the precise identification of reflection points along the probe from the start to the end. The measurement resolution of a TDR system depends on both the signal sampler and the length of the probe. In cases with limited hardware performance, the probe length can be extended through customized designs such as spiral or zigzag patterns. This study compares the performance of three different probe types, including a two-rod probe (N2), three-rod probe (N3), and two-rod spiral probe (S2), in measuring changes in water surface and sand bed elevations. Figure 9 presents the TDR monitoring results of changing bed elevation using three probes.

It can be seen from Figure 9 that the measuring errors of three types of probes are within ±10%, except for 3.0 cm cases. As the sand layer thickness increases, the error percentage decreases across all the probes. When the embedded length is insufficient, it is difficult to clearly identify reflecting points at the end of probes, which undermines the monitoring consistency. Furthermore, the spiral probe exhibits the most significant improvement in accuracy compared to the other two probes. The mean average relative error (MARE), which is equal to the ratio of absolute difference between TDR measurement and true value over true value, has been calculated for performance evaluations. The calculated MAREs are 11.3% for the N2 probe, 9.1% for the N3 probe, and 6.9% for the S2 probe. The spiral probe outperforms the N2 and N3 probes as it processes a much longer sensing length. The three-rod probe achieves better resolution than the two-rod probe, as it is structurally close to a coaxial cable and exhibits stronger anti-interference capabilities in signal transmission.

**Figure 10:**
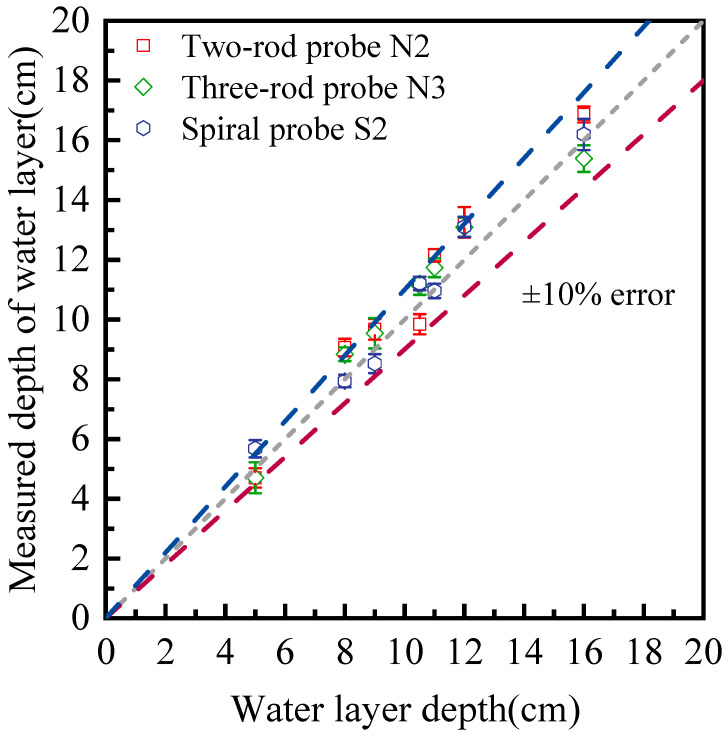
Measurement of water depth using different types of probes.

The results for water depth detection using three different types of probes are presented in Figure 10. A high consistency exists between TDR measurements and true values, and the measuring accuracy for all the probes lies within ±10%. Further analysis of measurement errors shows that the MARE is 8.6% for the N2 probe, 7.4% for the N3 probe, and 6.0% for the S2 spiral probe at room temperature. It is clearly shown the customized spiral probe outperforms the three-rod and two-rod probes in water depth measurement. It is also demonstrates that the TDR system is able to continuously monitor water level fluctuations with a high reliability.

**Figure 11:**
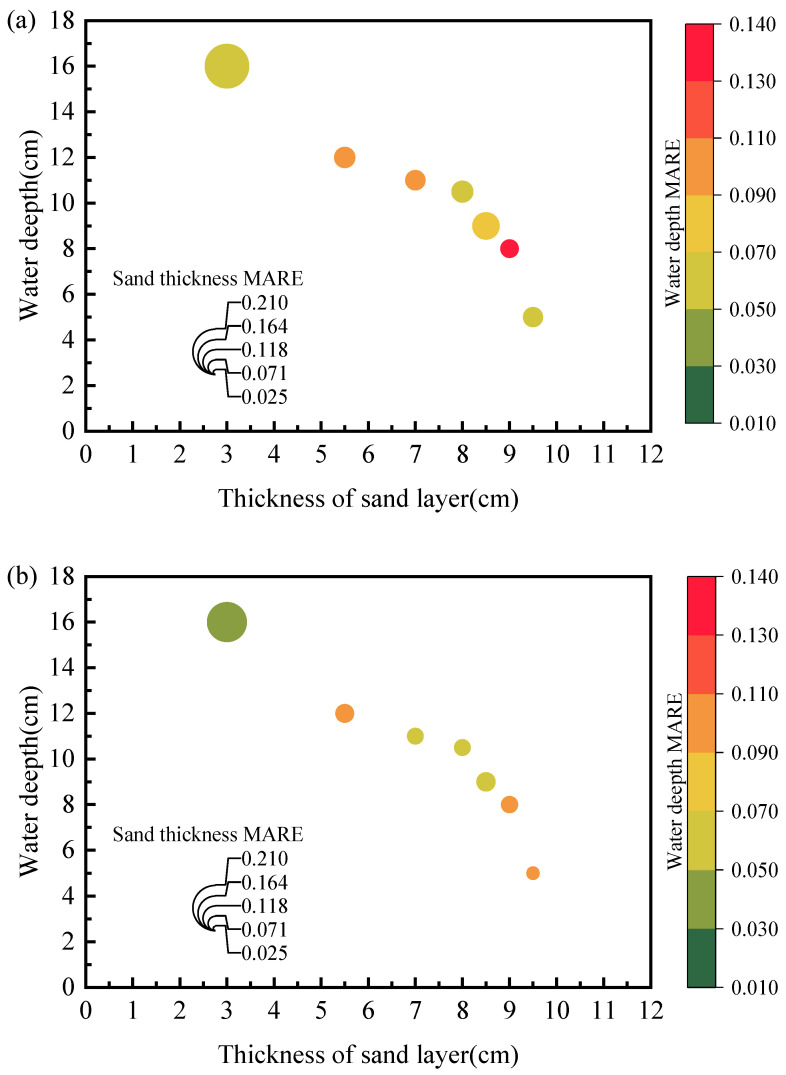

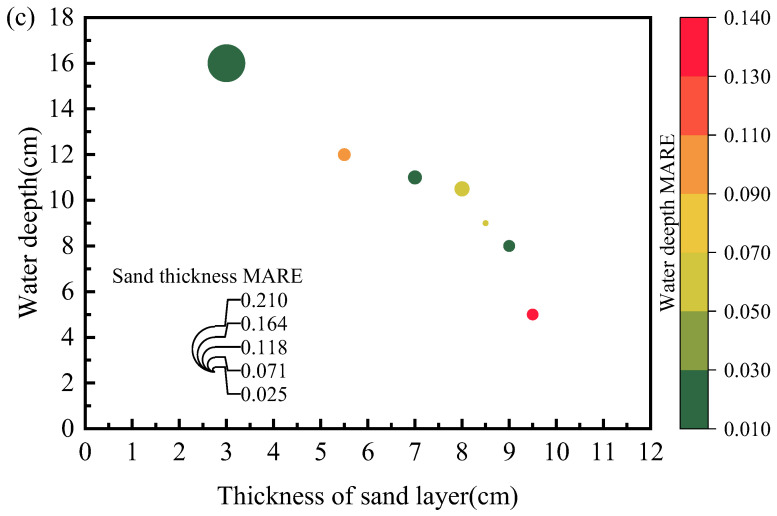
Comparison of MAREs over different water levels and embedding sand bed thicknesses using: (**a**) N2 probe; (**b**) N3 probe; (**c**) S2 probe.

Figure 11 further compares the MARE between TDR measurements and true values with three different probe configurations. The size of data spheres represents the average relative error in the measured thickness of the sandy soil layer, i.e., larger diameters indicate greater average relative errors. The measurements were acquired by assigning different combinations of water depth and sand bed elevation. The embedding sand layer thickness ranges from 3.0 cm to 9.5 cm, while the water depth varies between 5.0 cm to 16.0 cm. It should be noted the analysis is based on lab-scale tests using our customized probes, and field implementations require a redesign of longer probes, which would increase the detection accuracy. The associated signal attenuation could be compensated by proper adjusting of wire spacing and coating materials. It has been shown that, by proper selections of coating materials and coating thickness, the signal attenuation could be better addressed. Compared to the two-rod probe (Figure 11a) and three-rod probe (Figure 11b), the spiral probe showed a significant improvement in MARE as the embedding sand layer thickness decreased (see Figure 11c). For measurements over different water depths, the two-rod probe exhibited more pronounced errors than the other two types of probes.

### 4.3. Detections on Suspended Sediment Concentration

**Figure 12:**
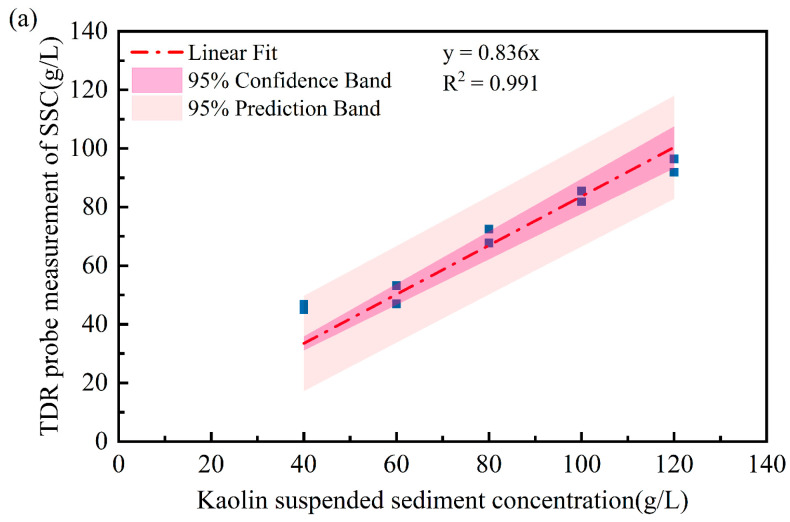

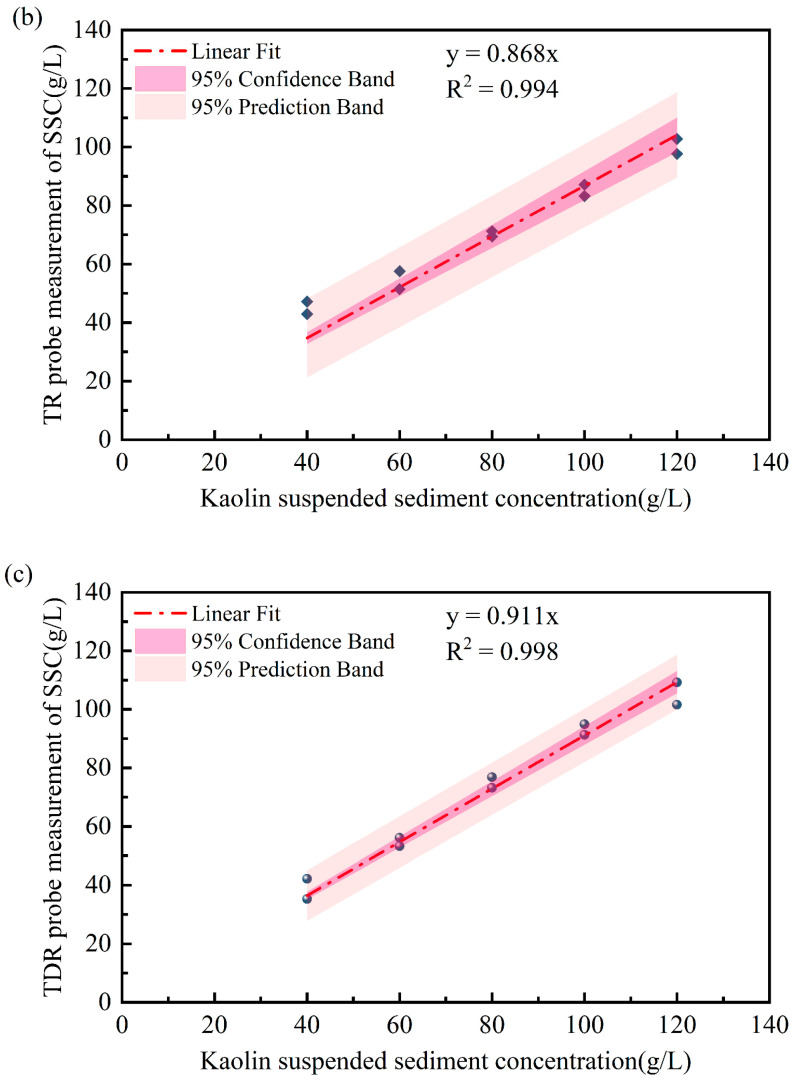
TDR measurements under varying SSC conditions using different probe types: (**a**) N2 probe; (**b**) N3 probe; (**c**) S2 probe.

The performance of the TDR system is further evaluated under different SSC conditions by mixing kaolinite clay with deionized water to prepare target suspensions ranging from 40 g/L to 120 g/L. To maintain a uniform suspended sediment distribution, an automatic stirrer has been utilized throughout the experiments. Figure 12 presents the correlation between prepared SSC values and TDR measurements using three different probes.

A linear regression is also provided between TDR measurements and true SSC values. There is a strong correlation existing for all the TDR probes. In particular, the SSCs measured with the spiral probe showed the lowest errors compared to the two-rod and three-rod probes. The concentrations measured by the three probes were lower than the prepared kaolin suspension concentration, primarily due to particle sedimentation and stratification in high-concentration kaolin suspensions. Even with rotational stirring to ensure uniform particle distribution, sedimentation and stratification remained unavoidable. Both the two-rod and spiral-shaped probes feature structural supports that stabilize the metal rods but reduce the contact area between the rods and suspended particles, thereby affecting concentration measurements.

Compared to the two-rod straight probe, the three-rod probe connects the central rod to the coaxial cable’s positive electrode while the outer two rods link to the shielding mesh. This configuration more closely resembles the structure of a coaxial cable, enabling more complete electromagnetic wave reception and transmission while minimizing signal leakage. The spiral structure extends the probe’s metal rod, granting the helical probe higher spatial resolution and sensitivity. Consequently, its measurement performance surpasses that of the other two probes.

## 5. Conclusions

This paper presents an innovatively designed TDR probe with data interpretation methodology to detect multiple hydrological parameters associated with flooding and scouring events in real time. The proposed TDR system is able to continuously monitor multiple information from a single reflective signal in the laboratory, including water surface, bed elevation, and SCC, which greatly reduces the installation and maintenance costs. The probe configuration has been improved from conventional spiral probes by integrating a spiral blade structure with a central support rod to form the metallic conductor support. The entire structure is fabricated using a 3D printer with two parallel signal transmission wires embedded in grooves along the outer edges of spiral blades, which achieves balances between easy installations in hard soils and detecting accuracy in different scenarios.

The proposed TDR system has been validated in the laboratory considering temperature-related corrections on water dielectric constants. The predicted error in sand layer thickness without considering the effect of temperature on water’s dielectric constant ranges between 10% and 15%. However, after applying temperature-dependent corrections, the error remains within 10% at both 24.1 °C and 35.3 °C. Based on the dielectric mixing model of the soil–water system, it is able to accurately extract different interfaces and electric properties of media from a single waveform. Accuracy analysis of water depth measurement at 20 °C showed MAREs of 8.6% for the N2 probe, 7.4% for the N3 probe, and 6.0% for the S2 helical probe. For sand layer thickness measurements at room temperature, the calculated MAREs were 11.3% for the N2 probe, 9.1% for the N3 probe, and 6.9% for the S2 probe. Compared to conventional two-rod and three-rod probes, the spiral-shaped probe features an improved spatial resolution from the helical design and a higher measuring accuracy across all the tests with MARE within 10%. The above study was conducted in laboratory conditions, and field implementations should be performed to better illustrate its performance. It should be noted the system may not be sensitive to low SSC suspensions, as the variation in electric properties is insignificant and environmental disturbance would be difficult to fully eliminate.

## Data Availability

The data cannot be made publicly available upon publication because no suitable repository exists for hosting data in this field of study. The data that support the findings of this study are available upon reasonable request from the authors.
